# Impact of Previous Coronary Artery Bypass Grafting on Clinical Outcomes in Patients With Acute Myocardial Infarction: A Systematic Review and Meta-Analysis

**DOI:** 10.7759/cureus.92383

**Published:** 2025-09-15

**Authors:** Mullapudi Lokesh, Anastasia Postoev, Loiy Naser Alsarkhi, Yousef Aqel, Iman Gillani, Rana M Ahzam, Calvin R Wei, Neelum Ali

**Affiliations:** 1 Medicine and Surgery, Gomel State Medical University, Gomel, BLR; 2 Internal Medicine, Caribbean Medical University, Willemstad, CUW; 3 Internal Medicine, Hamad Medical Corporation, Doha, QAT; 4 Medicine, Hamad Medical Corporation, Doha, QAT; 5 Medical Education, Sheikh Khalifa Bin Zayed Al-Nahyan University, Lahore, PAK; 6 Internal Medicine, Combined Military Hospital (CMH) Lahore Medical College and Institute of Dentistry, Lahore, PAK; 7 Research and Development, Shing Huei Group, Taipei, TWN; 8 Internal Medicine, University of Health Sciences, Lahore, PAK

**Keywords:** coronary artery bypass grafting, meta-analysis, mortality, myocardial infarction, stemi

## Abstract

This systematic review and meta-analysis examined the impact of previous coronary artery bypass grafting (CABG) on clinical outcomes in patients presenting with acute myocardial infarction (AMI). A comprehensive literature search was conducted across PubMed/MEDLINE, Embase, Cochrane Central Register of Controlled Trials (CENTRAL), and Web of Science from January 2010 to August 2025, following Preferred Reporting Items for Systematic Reviews and Meta-Analyses (PRISMA) guidelines. Observational studies comparing outcomes between patients with AMI with and without prior CABG history were included. Two independent reviewers performed study selection, data extraction, and quality assessment using the Newcastle-Ottawa Scale (NOS). Statistical analyses employed random-effects models using RevMan 5.4 (The Nordic Cochrane Centre, Copenhagen, Denmark) and R software (R Foundation for Statistical Computing, Vienna, Austria). Nine studies comprising patients with ST-segment elevation myocardial infarction (STEMI) and non-ST-segment elevation myocardial infarction (NSTEMI) were included in the final analysis. The pooled analysis demonstrated that previous CABG history was not significantly associated with all-cause mortality in the overall AMI population (relative risk (RR): 1.06, 95% confidence interval (CI): 0.97-1.16), although considerable heterogeneity was observed (I² = 87%). Subgroup analysis revealed that patients with STEMI with prior CABG had a 16% higher mortality risk compared to CABG-naïve patients (RR: 1.16, 95% CI: 1.12-1.20), while patients with NSTEMI showed a non-significant 6% increase (RR: 1.06, 95% CI: 0.95-1.19). No significant difference was found in major adverse cardiac events (MACE) between groups (RR: 0.98, 95% CI: 0.85-1.12). Meta-regression identified age, hypertension prevalence, and CABG prevalence as significant contributors to between-study heterogeneity. These findings suggest that prior CABG may confer increased mortality risk specifically in patients with STEMI, although limited study numbers in subgroup analyses warrant cautious interpretation and highlight the need for larger targeted investigations.

## Introduction and background

Acute myocardial infarction (AMI), encompassing both ST-segment elevation myocardial infarction (STEMI) and non-ST-segment elevation myocardial infarction (NSTEMI), represents a leading cause of cardiovascular morbidity and mortality worldwide, affecting over seven million individuals annually [1]. Despite significant advances in acute coronary syndrome management, including primary percutaneous coronary intervention (PCI) and contemporary medical therapy, substantial variations in clinical outcomes persist across different patient populations [2]. Among these populations, patients with a history of previous coronary artery bypass grafting (CABG) represent a particularly complex subgroup with unique pathophysiological characteristics and potentially distinct risk profiles.

The prevalence of patients with prior CABG presenting with subsequent AMI has been increasing as the population of post-surgical patients continues to age and develop progressive coronary artery disease [3]. These patients typically present with more complex coronary anatomy, advanced atherosclerotic burden, and multiple comorbidities compared to their CABG-naïve counterparts [4]. The pathophysiology underlying subsequent MI in post-CABG patients involves both native vessel progression and graft-related complications, including saphenous vein graft degeneration, which occurs in approximately 50% of grafts within 10 years of surgery [5].

Current evidence regarding the prognostic implications of previous CABG in patients with AMI remains conflicting and fragmented. Several observational studies have suggested that patients with prior CABG experience worse short-term and long-term outcomes following AMI, with reported in-hospital mortality rates ranging from 4.8% to 12.2% compared to 4.3% to 8.8% in CABG-naïve patients [6,7]. However, contemporary analyses from regional STEMI systems have demonstrated more favorable outcomes, suggesting that modern reperfusion strategies and medical management may have mitigated some of the historical disadvantages associated with prior CABG [8].

The complexity of managing AMI in post-CABG patients extends beyond immediate mortality outcomes. These patients face unique challenges in achieving optimal reperfusion, with culprit lesions occurring in native vessels in approximately 42% of cases and in bypass grafts in the remainder [9,10]. The choice of revascularization strategy, whether targeting the native vessel or the graft, significantly influences procedural success rates and clinical outcomes [11]. Furthermore, the temporal relationship between the original CABG procedure and subsequent MI appears to modify risk, with patients experiencing AMI within the first year after CABG demonstrating different risk profiles compared to those with a remote surgical history [12].

Despite the clinical importance of this population, no comprehensive systematic synthesis of the available evidence has been conducted to definitively establish the impact of previous CABG on AMI outcomes. The heterogeneity in study populations, outcome definitions, and follow-up periods across individual studies necessitates a rigorous meta-analytic approach to provide robust evidence for clinical decision-making. Understanding these outcomes is crucial for risk stratification, patient counseling, and optimizing treatment strategies in this high-risk population.

Therefore, we conducted this systematic review and meta-analysis to comprehensively evaluate the effect of previous CABG history on adverse outcomes in patients admitted with STEMI or NSTEMI, with the aim of providing evidence-based insights to guide clinical practice and inform future research priorities.

## Review

Methodology

Literature Search

A comprehensive systematic literature search was conducted in accordance with the Preferred Reporting Items for Systematic Reviews and Meta-Analyses (PRISMA) guidelines [13]. We searched multiple electronic databases, including PubMed/MEDLINE, Embase, Cochrane Central Register of Controlled Trials (CENTRAL), and Web of Science, from January 1, 2010, to August 10, 2025. The search strategy was developed using a combination of Medical Subject Headings (MeSH) terms and free-text keywords. The search strategy included the following key terms and their variations: "coronary artery bypass grafting," "CABG," "coronary artery bypass surgery," "myocardial infarction," "MI," "acute coronary syndrome," "STEMI," "NSTEMI," "mortality," "death," "clinical outcomes," and "major adverse cardiac events." Boolean operators (AND, OR) were used to combine search terms appropriately. The complete search strategy for each database was documented and is available in the supplementary materials. No language restrictions were applied during the initial search. The reference list of all included articles was manually screened to identify additional studies.

Study Selection

Two independent reviewers screened all titles and abstracts retrieved from the literature search using predefined inclusion and exclusion criteria. Full-text articles were obtained for all potentially eligible studies identified during the initial screening phase. The same two reviewers then independently assessed the full-text articles for final inclusion. Disagreements between reviewers were resolved through discussion, and when consensus could not be reached, a third reviewer was consulted for final arbitration. The selection process was documented using a PRISMA flow diagram showing the number of studies identified, screened, assessed for eligibility, and included in the final analysis.

Studies were considered eligible for inclusion if they were observational studies (cohort studies and case-control studies) that included patients with acute myocardial infarction (STEMI or NSTEMI) and compared clinical outcomes between patients with and without a prior history of coronary artery bypass grafting. Eligible studies were required to report at least one of the following outcomes: all-cause mortality, cardiovascular mortality, or major adverse cardiac events (MACE). Only studies published in peer-reviewed journals were included to ensure adequate methodological rigor and peer review process.

Studies were excluded if they were case reports, case series, editorials, letters, or review articles that did not provide original research data. When multiple studies reported on overlapping patient populations from the same database or registry, we included only the study with the largest sample size.

Data Extraction

Data extraction was performed independently by two reviewers using a standardized, pre-piloted data extraction form. The data extraction form was developed based on the Cochrane Handbook recommendations and was tested on a sample of five studies before full implementation. Any discrepancies in extracted data were resolved through discussion between the two reviewers, with involvement of a third reviewer when necessary. Data extraction included author, year, region, sample size, and participant characteristics.

Quality Assessment

The methodological quality of included studies was assessed independently by two reviewers using the Newcastle-Ottawa Scale (NOS) for observational studies [14]. The NOS evaluates studies across three domains: selection of study groups (4 points), comparability of groups (2 points), and ascertainment of outcome (3 points), with a maximum possible score of 9 points. Studies scoring 7-9 points were considered high quality, 5-6 points moderate quality, and 0-4 points low quality.

Data Analysis

Statistical analyses were performed using Review Manager (RevMan) version 5.4 (The Nordic Cochrane Centre, Copenhagen, Denmark) and R statistical software version 4.3.0 (R Foundation for Statistical Computing, Vienna, Austria) with the "meta" and "metafor" packages. All analyses followed random-effects models using the DerSimonian-Laird method due to anticipated clinical and methodological heterogeneity between studies.

Primary analysis involved pooled estimation of relative risk (RR) with 95% confidence intervals (CI) for all-cause mortality comparing patients with and without prior CABG history.

Assessment of heterogeneity was conducted using the Chi-squared test (significance level p < 0.10) and quantified using the I² statistic. Heterogeneity was interpreted as follows: I² ≤ 25% (low), 26%-50% (moderate), 51%-75% (substantial), and >75% (considerable). When substantial heterogeneity was detected (I² > 50%), we explored potential sources through subgroup and meta-regression analyses.

Meta-regression analysis was performed to investigate the relationship between study-level characteristics and treatment effects. Variables examined included mean age, proportion of male patients, prevalence of diabetes mellitus, hypertension, and hyperlipidemia. Meta-regression was conducted using restricted maximum likelihood estimation with the Knapp-Hartung adjustment for small sample sizes. Statistical significance was defined as p < 0.05 for all analyses except heterogeneity testing (p < 0.10). Results are presented as relative risks with 95% confidence intervals. We were unable to assess publication bias because the number of studies was less than 10.

Results

**Figure 1:**
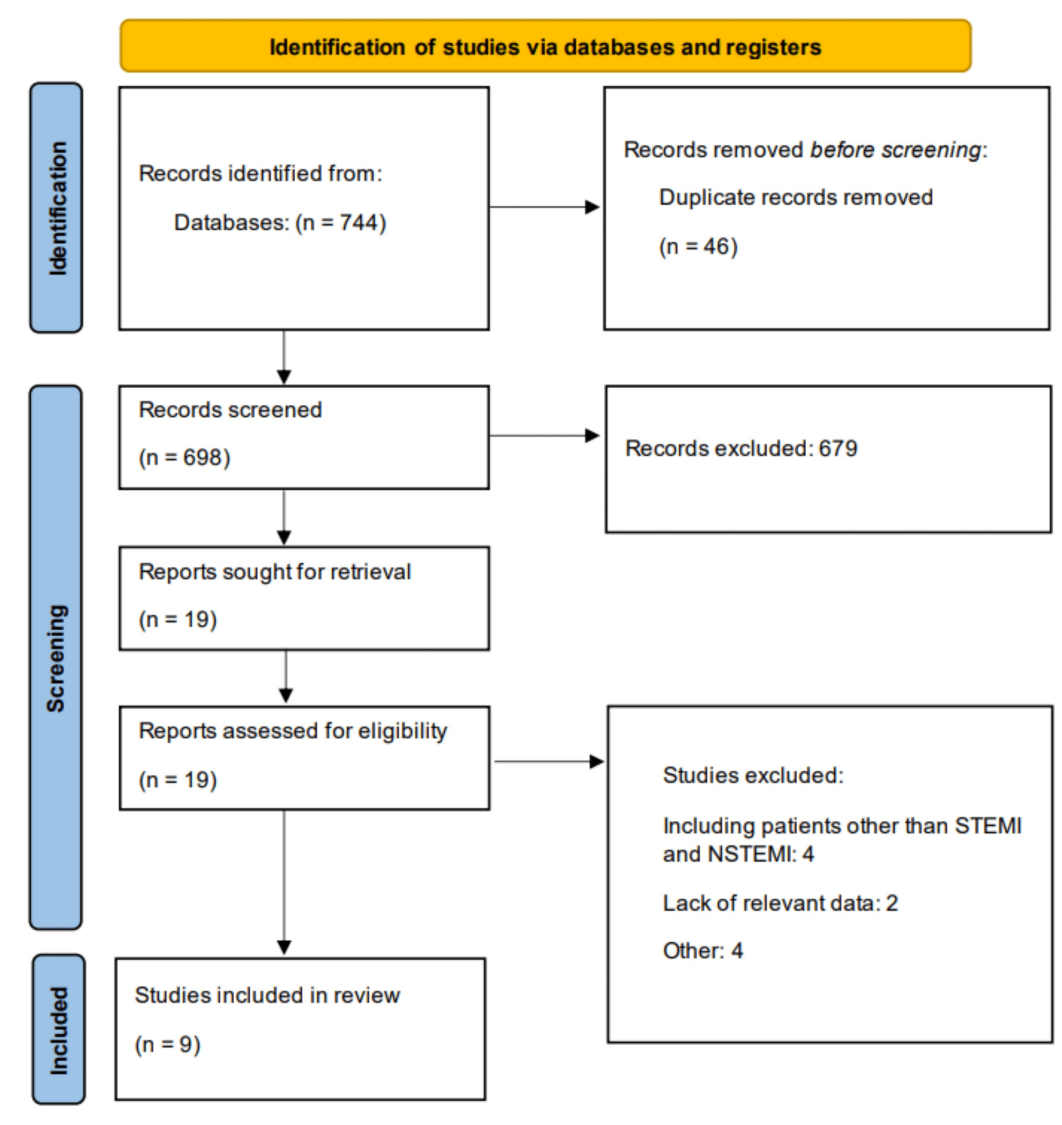
PRISMA flowchart PRISMA: Preferred Reporting Items for Systematic Reviews and Meta-Analyses, STEMI: ST-segment elevation myocardial infarction, NSTEMI: non-ST-segment elevation myocardial infarction

Figure 1 presents the PRISMA flowchart of the study selection process. A total of 744 studies were identified through database searching, of which 698 remained after removing duplicates. Following full-text screening of 19 studies, nine studies met the eligibility criteria and were included in the meta-analysis. Table 1 summarizes the characteristics of the included studies. The total sample size across studies ranged from fewer than 200 to more than 2.7 million participants. Some studies exclusively evaluated patients with STEMI, others with NSTEMI, while one study included a mixed population of both STEMI and NSTEMI. The proportion of patients undergoing CABG compared with those managed without CABG varied considerably across studies, highlighting differences in study design, patient populations, and clinical practice patterns. Table 2 presents the quality assessment of the included studies.

**Table 1:** Characteristics of the included studies CABG: coronary artery bypass grafting, STEMI: ST-segment elevation myocardial infarction, NSTEMI: non-ST-segment elevation myocardial infarction, MI: myocardial infarction, NR: not reported

Author	Type of MI	Groups	Sample size	Age (years)	Male (number)	Hypertension (number)	Diabetes (number)	Hyperlipidemia (number)
Dhaduk et al. (2023) [15]	NSTEMI	CABG	133,691	72.39	91,946	89,724	44,520	NR
		No CABG	1,311,854	68.57	745,929	848,821	344,362	
Gharacholou et al. (2020) [16]	STEMI	CABG	305	70.4	218	251	107	249
		No CABG	3,950	64.3	2,065	1,850	550	1,801
Hart et al. (2025) [17]	STEMI	CABG	35	68	27	31	16	29
		No CABG	801	60	576	566	250	491
Kim et al. (2010) [18]	NSTEMI	CABG	8,790	72	6,153	7,456	4,317	6,937
		No CABG	38,767	66	22,834	27,474	38,767	20,265
Lemor et al. (2020) [19]	STEMI + NSTEMI	CABG	42,147	82	29,081	38,480	20,527	32,790
		No CABG	253,915	83	118,071	216,843	90,902	161,998
Shoaib et al. (2021) [20]	NSTEMI	CABG	25,296	74	20,047	15,943	10,412	11,875
		No CABG	262,362	71	163,721	141,155	62,559	87,740
Pancholy et al. (2021) [6]	STEMI	CABG	110,066	72	78,065	76,846	43,740	NR
		No CABG	2,600,309	64	1,685,751	1,550,826	742,983	
Tabowei et al. (2025) [21]	STEMI	CABG	48,510	NR	NR	NR	NR	NR
		No CABG	503,514					
Welsh et al. (2010) [7]	STEMI	CABG	128	69	110	90	32	NR
		No CABG	5,617	61	4,311	2,749	187	

**Table 2:** Quality assessment of the included studies

Author	Selection	Comparability	Assessment	Overall
Dhaduk et al. (2023) [15]	3	2	2	Good
Gharacholou et al. (2020) [16]	4	2	2	Good
Hart et al. (2025) [17]	3	2	2	Good
Kim et al. (2010) [18]	3	1	3	Good
Lemor et al. (2020) [19]	4	2	3	Good
Shoaib et al. (2021) [20]	4	2	3	Good
Pancholy et al. (2021) [6]	3	2	2	Good
Tabowei et al. (2025) [21]	3	1	2	Fair
Welsh et al. (2010) [7]	4	2	2	Good

Mortality

**Figure 2:**
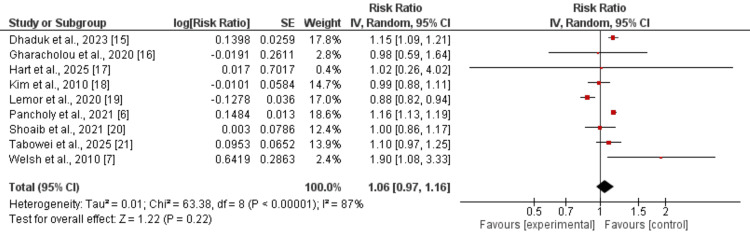
Association of CABG with mortality References [6,7,15-21] CABG: coronary artery bypass grafting

Nine studies were included to evaluate the impact of previous CABG on mortality in patients with MI, with pooled results presented in Figure 2. The overall analysis showed that a history of CABG was not significantly associated with all-cause mortality (RR: 1.06, 95% CI: 0.97-1.16), although substantial heterogeneity was observed (I² = 87%). Subgroup analysis by type of MI revealed that, in patients with STEMI, prior CABG was associated with a 16% higher risk of mortality (RR: 1.16, 95% CI: 1.12-1.20). In contrast, among patients with NSTEMI, the risk of mortality was 6% higher in those with prior CABG, but this difference did not reach statistical significance (RR: 1.06, 95% CI: 0.95-1.19).

MACE

**Figure 3:**
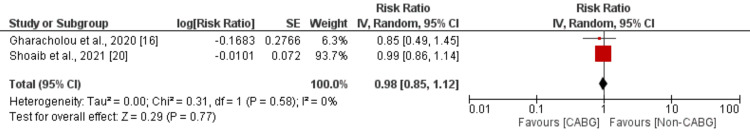
Association of CABG with MACE References [16,20] CABG: coronary artery bypass grafting, MACE: major adverse cardiac event

Two studies were included to compare the risk of MACE between subjects who had a history of CABG and those without a history of CABG, and the results are shown in Figure 3. Pooled analysis of the two studies showed no significant difference in the risk of MACE between the two groups (RR: 0.98, 95% CI: 0.85-1.12). No significant heterogeneity was reported among the study results (I^2^ = 0%). 

Meta-Regression for Mortality

Table 3 presents the findings of the meta-regression. In the meta-regression analysis, age and hypertension emerged as significant contributors to mortality among patients with myocardial infarction, with studies enrolling older populations or those with a higher prevalence of hypertension demonstrating worse outcomes. In contrast, the proportion of male patients, diabetes, and hyperlipidemia did not significantly explain between-study variability in mortality, despite their well-recognized role as individual-level risk factors. Interestingly, a higher prevalence of coronary artery bypass grafting (CABG) within study cohorts was associated with mortality, suggesting patients may be at higher risk of mortality. Overall, these findings indicate that study-level variations in age, hypertension, and CABG prevalence may account for a substantial proportion of heterogeneity in mortality outcomes following myocardial infarction.

**Table 3:** Meta-regression CABG: coronary artery bypass grafting

Variable	Coefficient	P-value	Heterogeneity accounted for
Age	0.0149	0.001	76.80%
Male	0.017	0.2828	48.70%
Diabetes	-0.0024	0.3029	0%
Hypertension	0.0087	0.0034	73.27%
Hyperlipidemia	-0.0043	0.325	68.93%
CABG	0.0184	0.0284	46.79%

Discussion

In this systematic review and meta-analysis, we examined the impact of prior coronary artery bypass grafting (CABG) on clinical outcomes in patients presenting with myocardial infarction (MI). The pooled analysis demonstrated that a history of CABG was not significantly associated with all-cause mortality in the overall MI population, although considerable heterogeneity was observed across studies. Subgroup analyses provided further insights, showing an elevated risk of mortality in patients with ST-segment elevation MI (STEMI) and a non-significant trend toward increased risk in those with non-ST-segment elevation MI (NSTEMI).

The pathophysiology of MI in post-CABG patients involves both native vessel progression and graft-related complications. Native coronary artery disease continues to progress following CABG, with annual rates of significant stenosis development ranging from 5%-10% [22]. Additionally, saphenous vein grafts are particularly susceptible to accelerated atherosclerosis, with approximately 10%-20% developing occlusion within the first year and up to 50% showing significant stenosis by 10 years post-surgery [23]. Arterial grafts, while demonstrating superior long-term patency, are not immune to disease progression, particularly at anastomotic sites [24]. 

Our findings align with several observational studies that have reported mixed outcomes in post-CABG patients presenting with acute MI, although direct comparisons are limited by the small number of studies in our subgroup analyses. The Global Registry of Acute Coronary Events (GRACE) registry analysis by Eagle et al. showed that prior CABG was an independent predictor of mortality in acute coronary syndrome patients, with the effect being most pronounced in those presenting with STEMI [25]. However, these findings must be interpreted cautiously, given that our STEMI subgroup analysis was based on only five studies [6,7,16,17,21], limiting the robustness of this comparison.

The scarcity of studies specifically examining NSTEMI outcomes (only three studies in our analysis) [15,18,20] is particularly concerning given that NSTEMI represents the majority of acute coronary syndrome presentations in the contemporary era. This limitation significantly restricts our ability to draw definitive conclusions about the impact of prior CABG on NSTEMI outcomes.

However, some studies have reported contradictory findings. The analysis by Gersh et al. from the GUSTO-I trial suggested that while patients with prior CABG had higher baseline risk profiles, their adjusted mortality outcomes were not significantly different from those without prior surgery [26]. These discrepancies may be explained by differences in study populations, definitions of outcomes, and analytical approaches, highlighting the importance of our comprehensive meta-analytic approach.

The management of MI in patients with prior CABG presents unique challenges that require careful consideration. Primary percutaneous coronary intervention (PCI) remains the preferred reperfusion strategy; however, the technical complexity is significantly increased [27]. Interventional cardiologists must navigate complex anatomy, including determining whether the culprit lesion is in a native vessel, bypass graft, or at an anastomotic site. This complexity often results in longer door-to-balloon times, which may contribute to the worse outcomes observed in our analysis [28].

Several important limitations must be acknowledged when interpreting our findings. The substantial heterogeneity (I² = 87%) observed in our mortality analysis warrants careful interpretation. This heterogeneity likely reflects differences in study populations, definitions of outcomes, follow-up duration, and treatment protocols across the included studies. Our meta-regression analysis identified age, hypertension prevalence, and CABG prevalence as significant contributors to between-study variability, suggesting that these factors should be carefully considered when interpreting results from individual studies.

A critical limitation of our analysis is the relatively small number of studies included in the subgroup analyses by MI type. Only five studies provided data specifically for patients with STEMI, while merely three studies assessed outcomes in patients with NSTEMI. This limited number of studies substantially reduces the statistical power of our subgroup analyses and increases the uncertainty around the effect estimates. The finding of increased mortality risk in patients with STEMI (RR: 1.16, 95% CI: 1.12-1.20) should be interpreted cautiously, given this limited sample size, despite appearing statistically significant. Similarly, the non-significant trend toward increased mortality in patients with NSTEMI (RR: 1.06, 95% CI: 0.95-1.19) may reflect insufficient power to detect a clinically meaningful difference rather than a true absence of effect.

The paucity of studies specifically examining NSTEMI outcomes represents a significant gap in the literature, as NSTEMI accounts for approximately 70% of all MI presentations in contemporary practice [29]. This underrepresentation may limit the generalizability of our findings to the broader MI population and highlights the need for future studies to specifically examine outcomes in patients with NSTEMI with prior CABG.

The time interval between CABG and MI presentation was not consistently reported across studies, which represents an important limitation. Early postoperative MI (within 30 days of CABG) has different implications compared to late presentation (years after surgery), as the former may be related to perioperative complications, while the latter typically reflects disease progression [30].

Future research should focus on several key areas to better understand and optimize outcomes in post-CABG patients with MI. First, and most urgently, there is a critical need for larger studies specifically examining outcomes in patients with STEMI and NSTEMI with prior CABG. The current evidence base, with only five studies for STEMI and three for NSTEMI, is insufficient to draw definitive conclusions about differential outcomes by MI type. Large prospective registries specifically designed to capture the unique characteristics of this population are needed, with adequate sample sizes to enable robust subgroup analyses by MI type. Such studies should include detailed information about graft characteristics, time from surgery, and contemporary treatment protocols.

Second, the development of risk stratification tools specifically for post-CABG patients with MI could help clinicians make more informed treatment decisions. These tools should incorporate both traditional cardiovascular risk factors and CABG-specific variables such as graft type, age, and patency status.

Third, randomized controlled trials comparing different reperfusion strategies in post-CABG patients with MI are needed. Current guidelines are largely based on expert consensus and observational data, highlighting the need for higher-quality evidence to guide clinical practice.

## Conclusions

This systematic review and meta-analysis demonstrates that previous coronary artery bypass grafting history does not significantly increase overall mortality risk in patients with acute myocardial infarction. However, subgroup analysis reveals important distinctions by MI type, with patients with STEMI showing significantly elevated mortality risk, while patients with NSTEMI demonstrate non-significant trends toward increased mortality. The substantial heterogeneity observed across studies and the limited number of investigations in subgroup analyses necessitate cautious interpretation of these findings. The paucity of studies specifically examining NSTEMI outcomes represents a critical knowledge gap, particularly given NSTEMI's predominance in contemporary practice. Future research should prioritize large-scale prospective studies with adequate sample sizes to definitively establish differential outcomes by MI type. These findings highlight the need for tailored risk stratification and management strategies in post-CABG patients presenting with acute myocardial infarction, particularly those with STEMI.
